# First diagnosis of psychosis in the prison: results from a data-linkage study

**DOI:** 10.1192/bjo.2019.74

**Published:** 2019-10-14

**Authors:** Nabila Z. Chowdhury, Olayan Albalawi, Handan Wand, Armita Adily, Azar Kariminia, Stephen Allnutt, Grant Sara, Kimberlie Dean, Julia Lappin, Colman O'Driscoll, Luke Grant, Peter W. Schofield, David Greenberg, Tony Butler

**Affiliations:** PhD student, Kirby Institute, University of New South Wales, Australia; Tabuk University, Department of Statistics, Science Faculty, Saudi Arabia; Associate Professor, Kirby Institute, University of New South Wales, Australia; Research Fellow, Kirby Institute, University of New South Wales, Australia; Senior Lecturer, Kirby Institute, University of New South Wales, Australia; Forensic Psychiatrist and Conjoint Senior Lecturer, University of New South Wales, Australia; Director, InforMH, NSW Ministry of Health; and Clinical Associate Professor, University of Sydney Northern Clinical School, Australia; Associate Professor, Forensic Mental Health, School of Psychiatry, University of New South Wales, Australia; Psychiatrist, School of Psychiatry, University of New South Wales, Australia; Executive Director, Lifeline Australia; and Conjoint Lecturer, University of New South Wales, Australia; Assistant Commissioner, Corrections Strategy & Policy, Corrective Services NSW, Australia; FRACP Clinical Director, Neuropsychiatry Service, Hunter New England Local Health District; and Conjoint Professor, University of Newcastle, Australia; Director, New South Wales State-Wide Clinical Court Liaison Service, New South Wales Justice and Forensic Mental Health Network; and Conjoint Lecturer, University of New South Wales, Australia; Program Head, Justice Health Research Program, Kirby Institute, University of New South Wales, Australia

**Keywords:** First diagnosis of psychosis, prison, community, data-linkage, time to diagnosis

## Abstract

**Background:**

Psychosis is more prevalent among people in prison compared with the community. Early detection is important to optimise health and justice outcomes; for some, this may be the first time they have been clinically assessed.

**Aims:**

Determine factors associated with a first diagnosis of psychosis in prison and describe time to diagnosis from entry into prison.

**Method:**

This retrospective cohort study describes individuals identified for the first time with psychosis in New South Wales (NSW) prisons (2006–2012). Logistic regression was used to identify factors associated with a first diagnosis of psychosis. Cox regression was used to describe time to diagnosis from entry into prison.

**Results:**

Of the 38 489 diagnosed with psychosis for the first time, 1.7% (*n* = 659) occurred in prison. Factors associated with an increased likelihood of being diagnosed in prison (versus community) were: male gender (odds ratio (OR) = 2.27, 95% CI 1.79–2.89), Aboriginality (OR = 1.81, 95% CI 1.49–2.19), older age (OR = 1.70, 95% CI 1.37–2.11 for 25–34 years and OR = 1.63, 95% CI 1.29–2.06 for 35–44 years) and disadvantaged socioeconomic area (OR = 4.41, 95% CI 3.42–5.69). Eight out of ten were diagnosed within 3 months of reception.

**Conclusions:**

Among those diagnosed with psychosis for the first time, only a small number were identified during incarceration with most identified in the first 3 months following imprisonment. This suggests good screening processes are in place in NSW prisons for detecting those with serious mental illness. It is important these individuals receive appropriate care in prison, have the opportunity to have matters reheard and possibly diverted into treatment, and are subsequently connected to community mental health services on release.

**Declaration of interest:**

None.

High rates of psychosis and other mental disorders are common in prisoner populations worldwide.^1^ A meta-analysis of 33 588 prisoners covering 24 countries reported a pooled prevalence of psychosis of 3.6% in men and 3.9% in women.^2^ An Australian study reported that prisoners were 11.8 times more likely to report symptoms of psychosis than the general community.^3^ It is therefore important that strategies are in place to continue treatment from the community, screen for those with serious mental illness on entry to prison and ensure continuity of treatment on release from prison. In some cases, those with serious mental illness may have been missed by court diversion schemes aimed at deflecting those with psychiatric illness away from custody and into treatment programmes and therefore prison screening can provide a further opportunity to identify those requiring treatment. One study using the Prodromal Questionnaire among newly arrived male prisoners (*n* = 750) in England, found that 5% met the diagnostic criteria for a psychotic disorder with 3% having recently developed a first episode of psychosis (FEP).^4^ An Australian study using a psychosis screener found that 10.7% of male and 15.2% of female reception prisoners (screened within 24 h of admission to prison), and 4.2% of male and 5.7% female sentenced prisoners tested positive for symptoms of psychosis in the past 12 months.^5^ In a sample of over 3000 newly arrived prisoners at five prisons in England who were screened for mental illness within 3 days of reception psychiatric symptoms were highest during the first week in custody.^6^ However, poor resourcing in the face of the huge demand placed on prison mental health services often results in only the most severe cases being identified and treated.^7^

Prison has an important role to play in screening those with serious mental illness as untreated FEP has been shown to be a risk factor for offending.^8^ A systematic review of studies of homicide in those with psychosis found an association with a longer duration of untreated psychosis.^9^ A UK study reported that 9.6% of those with FEP had committed at least one act of serious aggression (weapon use, sexual assault or victim injury) during the period between the first onset of psychosis and contact with psychiatric services.^10^ However, studies have also showed that when confounding factors such as substance misuse are considered, the reported association between early psychosis-related factors and violence weakens.^11^ The current study describes those identified as having a first diagnosis of psychosis in NSW prisons, between July 2006 and December 2012, factors associated with a first diagnosis in prison and time to first diagnosis after entry into prison using data from a population-based linkage study.

## Method

### Study population

**Fig. 1:**
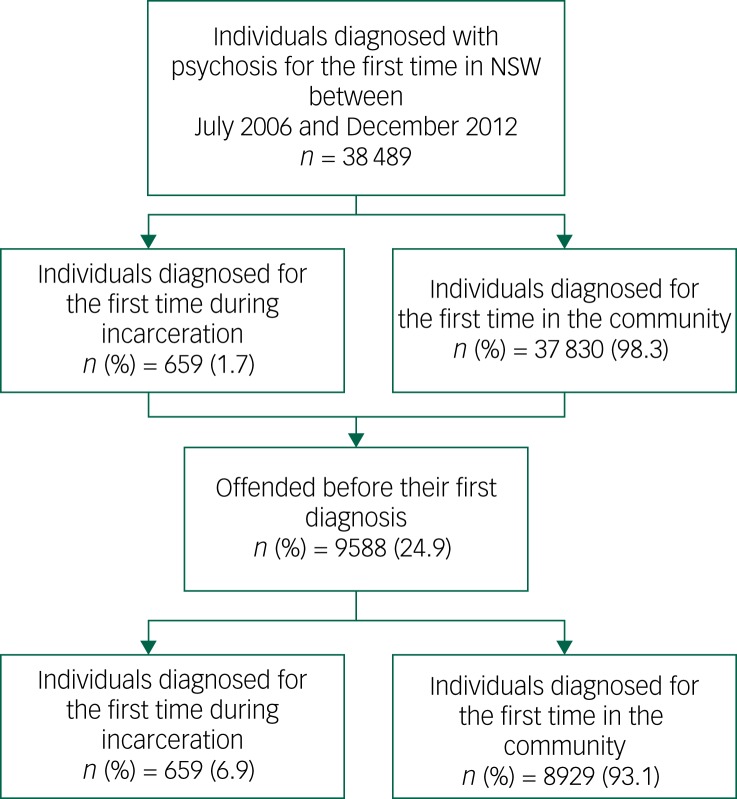
Flow chart of study population.

Our study population included all individuals who had a first diagnosis of psychosis between 1 July 2006 and 31 December 2012 in NSW, Australia. To identify this cohort, we first included all individuals in NSW with at least one public or private hospital admission episode or emergency department presentation in which a primary or additional diagnosis of psychosis was recorded in either the NSW Admitted Patients Data Collection (APDC) between July 2001 and December 2012, or in the NSW Emergency Patients Data Collection (EDDC) between June 2005 and December 2012 (Fig. 1). We then selected those who had their first diagnosis of psychosis between July 2006 and December 2012 and no recorded psychosis diagnosis for at least 5 years prior to this going back to July 2001 in either the APDC or EDDC. Additionally, we examined the NSW Mental Health Ambulatory data collection (MH-AMB) to determine whether any of the first-diagnosis group had any psychosis-related presentation in this collection between July 2006 and December 2012 and before the diagnosis dates determined from APDC or EDDC and if so, considered this to be the first diagnosis. We excluded those who had any psychosis-related presentation in MH-AMB before July 2006. Prison diagnoses and treatment episodes are recorded in the APDC, EDDC and the MH-AMB database.

NSW, New South Wales.

We divided this first-diagnosis group into two groups based on the setting at the time of diagnosis – prison and community (note that community refers to a diagnosis made in either a hospital or community mental health service setting). The prison group included all individuals who were diagnosed for the first time during incarceration identified from the Corrective Services NSW Offender Integrated Management System (OIMS).

Two subgroups of offenders were created from those with a first diagnosis of psychosis in prison and in the community who had offended at least once before their first diagnosis. These two groups were derived to determine the factors associated with a first diagnosis of psychosis in prison. We further examined time to diagnosis in prison from entry into prison.

### Definition of psychosis

Psychosis was identified according to ICD-9^12^ and ICD-10^13^ codes from the APDC and EDDC data-sets. Psychotic disorders were defined as: schizophrenia and related psychoses (F20, F22–F25, F28, F29 and 295), affective psychoses (F30.2, F31.2, F31.5, F32.3 F33.3, 296.8 and 296.9) and substance-related psychoses (F10.5, F11.5, F12.5, F13.5, F14.5, F15.5, F16.5, F17.5, F18.5, F19.5, 291 and 292). The EDDC also includes the Systematized Nomenclature of Medicine – Clinical Terms^14^ which were mapped to the ICD-10 codes.

### Data sources

In this study we used several administrative data collections from the NSW health and justice system.

To identify individuals with a first diagnosis of psychosis, we used two data collections: NSW APDC, which records all hospital admissions to public and private hospitals and day procedure centres in NSW and NSW EDDC, which covers all presentations to emergency departments in public hospitals in NSW. We extracted information on gender (men, women), Aboriginal and/or Torres Strait Islander (yes, no), psychosis type (schizophrenia and related psychoses, affective psychoses, substance-related psychoses), age at first diagnosis, marital status at the time of diagnosis (married including de facto, other, and unknown) and statistical local area (SLA) of residence from these data-sets. (A de facto relationship, under the Family Law Act 1975, is defined as a relationship between two people who are not legally married or related by family, lived together on a genuine domestic basis.) The ‘other’ category of marital status included those who were single, widowed, divorced or permanently separated from their partner.

We categorised age at the time of first diagnosis of psychosis in four groups (<25 years, 25–34 years, 35–44 years and >45 years). Socio-Economic Index for Area (SEIFA) categorises postcodes into ‘advantaged’, ‘disadvantaged’ and ‘unknown’ using the Index of Relative Socio-economic Advantage and Disadvantage developed by the Australian Bureau of Statistics.^15^ The lowest rank indicates the most disadvantaged area and the highest rank the most advantaged. Thus, it allowed the areas (SLAs) to be categorised into disadvantaged (SEIFA score 1–5) and advantaged (SEIFA score 6–10) areas.^15^ APDC was available from July 2001 to December 2012 and EDDC from January 2005 to December 2012.

We used NSW MH-AMB to identify those individuals selected from APDC and EDDC who presented to community mental health services. We collected information on date of contact from this data collection. This data collection was available from July 2001 to December 2012.

Offence records were derived from the NSW Bureau of Crime Statistics and Research's Re-offending Database. Criminal convictions were coded according to the Australian and New Zealand Standard Offence Classification (ANZSOC) and minor traffic infringements were excluded. We extracted data on principal offence, date of offence and offence type from this data-set. For analysis purposes we grouped offences into violent (ANZSOC codes 111–621) and non-violent (ANZSOC codes 711–1699). Data were available from July 2001 to June 2015.

Date of entry into prison, date of release from prison and information on previous episodes of incarceration was extracted from the Corrective Services NSW OIMS. These data were available from July 2001 to June 2014.

Linkage between the data-sets was performed by the Centre for Health Record Linkage using the probabilistic record linkage method. All the demographic data were collected at the time of the first psychosis diagnosis.

### Statistical analysis

We described characteristics at the time of a first diagnosis of psychosis and compared these characteristics by diagnosis setting (i.e. inside prison and in the community). Multivariate logistic regression models were used to identify factors associated with the first diagnosis of psychosis in prison for men and women separately. Time to diagnosis inside prison from entry into prison was summarised using a cumulative frequency plot and Kaplan–Meier survival plots and Cox proportional hazard regression models were used to determine the factors associated with early diagnosis during any prison episode for men and women separately. SAS Version 9·4 and Stata 10·0 was used to analyse the data.

### Ethics statement

The authors assert that all procedures contributing to this work comply with the ethical standards of the relevant national and institutional committees on human experimentation and with the Helsinki Declaration of 1975, as revised in 2008. Ethics approvals were obtained from the NSW Population & Health Services Human Research Ethics Committee or HREC (HREC/15/CIPHS/17); Justice Health and Forensic Mental Health Network HREC (G324/14); Corrective Services NSW Ethics Committee (D15/138715); and the NSW Aboriginal Health and Medical Research Council HREC (1089/15).

### Consent statement

This study examines de-identified population data that has been collected during routine clinical care. Project-specific person number was the only identifier of the linked data provided by the Centre for Health Record Linkage; the researchers had no access to other individual identifiers.

## Results

### Characteristics of those with a first psychosis diagnosis

A total of 38 489 people were identified as having their first diagnosis of psychosis between July 2006 and December 2012 in NSW of whom 55.6% were men and 6.5% were of Aboriginal heritage (Table 1). Around one-fifth (21.4%) were married, 41.0% were aged 45 years and above and over half (51.0%) were from disadvantaged areas based on the SEIFA classification. Almost two-thirds (63.1%) were diagnosed with schizophrenia and related psychoses.

**Table 1:** Characteristics of individuals with a first diagnosis of psychosis by setting (prison or community) in New South Wales, July 2006 to December 2012

Characteristics	Total (*n* = 38 489)	Diagnosed during prison episode (*n* = 659) (1.7%)	Diagnosed in the community (*n* = 37 830) (98.3%)	*P*
	*n* (%)	*n* (col %) (row %)	*n* (col %) (row %)	
Gender				
Women	17 082 (44.4)	84 (12.7) (0.5)	16 998 (44.9) (99.5)	<0.001
Men	21 407 (55.6)	575 (87.3) (2.7)	20 832 (55.1) (97.3)	
Aboriginal				
No	35 973 (93.5)	486 (73.7) (1.4)	35 487 (93.8) (98.6)	<0.001
Yes	2516 (6.5)	173 (26.3) (6.9)	2343 (6.2) (93.1)	
Psychosis type				
Schizophrenia and related psychoses	24 290 (63.1)	521 (79.1) (2.1)	23 769 (62.8) (97.9)	<0.001
Affective psychoses	6329 (16.4)	40 (6.0) (0.6)	6289 (16.6) (99.4)	
Substance-related psychoses	7870 (20.4)	98 (14.9) (1.2)	7772 (20.5) (98.8)	
Age at first diagnosis, years				
<25	7455 (19.3)	154 (23.4) (2.1)	7301 (19.3) (97.9)	<0.001
25–34	8035 (20.9)	233 (35.4) (2.9)	7802 (20.6) (97.1)	
35–44	7216 (18.7)	180 (27.3) (2.5)	7036 (18.6) (97.5)	
>45	15 783 (41.0)	92 (14.0) (0.6)	15 691 (41.5) (99.4)	
SEIFA				
Advantaged	16 141 (41.9)	70 (10.6) (0.4)	16 071 (42.5) (99.6)	<0.001
Disadvantaged	19 611 (51.0)	572 (86.8) (2.9)	19 039 (50.3) (97.1)	
Unknown	2737 (7.1)	17 (2.6) (0.6)	2720 (7.2) (99.4)	
Marital status				
Married (including de facto)	8247 (21.4)	116 (17.6) (1.4)	8131 (21.5) (98.6)	<0.001
Other^a^	22 425 (58.3)	440 (66.8) (2.0)	21 985 (58.1) (98.0)	
Missing/unknown	7817 (20.3)	103 (15.6) (1.3)	7714 (20.4) (98.7)	

SEIFA, Socio-Economic Indexes for Areas.

a. For example single, widowed, divorced, permanently separated.

Only 1.7% (659/38 489) of this group were diagnosed for the first time with psychosis during a prison episode with the rest (*n* = 37 830; 98.3%) diagnosed for the first time in the community. Overall, men were significantly more likely than women (2.7% *v.* 0.5%, *P*<0.001) to be diagnosed with psychoses for the first time in prison rather than in the community. Those who were of Aboriginal heritage were more likely (6.9% *v.* 1.4%, *P*<0.001) to be first diagnosed with psychosis in prison rather than in the community. People who had their first diagnosis in prison were more likely to have a diagnosis of schizophrenia and related psychosis (79.1% *v.* 62.9%, *P*<0.001) and were less likely to have a diagnosis of either affective psychoses or substance-related psychoses (6.0% *v.* 16.6%, *P*<0.001, and 14.9% *v.* 20.5%, *P*<0.001, respectively) than people whose first diagnosis was in the community.

A smaller proportion of those aged 45 years and above were diagnosed for the first time in prison rather than the community (13.9% *v.* 41.5%, *P*<0.001). Those living in a disadvantaged area prior to prison were more likely to be diagnosed in prison than in the community (86.8% *v.* 50.3%, *P*<0.001). Those of single marital status were more likely to be diagnosed in prison than the community (66.8% *v.* 58.1%, *P*<0.001).

### Offending characteristics of people with a first diagnosis of psychosis

Overall, 24.9% (9588/38 489) of those with a first diagnosis of psychosis had been convicted of an offence prior to the first diagnosis (Table 2). The median time from offence to diagnosis was 13.3 months (interquartile range (IQR) 2.67–37.68 months). Non-violent offending was higher than violent offending overall (64.8% *v*. 34.6%) and also in both subgroups of first-diagnosed individuals. Of those with a prior offending history, 6.9% (*n* = 659) were diagnosed for the first time with psychosis during a period of incarceration.

**Table 2:** Principal offence type before the first diagnosis by setting (prison (*n* = 659; 6.9%); community (*n* = 8929; 93.1%))

	Total (*n* = 9588)	Diagnosed during prison episode (*n* = 659) (6.9%)	Diagnosed in the community (*n* = 8 929) (93.1%)
Violent offence (ANZSOC code)	3321 (34.6)	261 (39.6)	3060 (34.3)
Acts intended to cause injury (211–299)	2493 (26.0)	166 (25.2)	2327 (26.1)
Dangerous or negligent acts (411–499)	451 (4.7)	18 (2.7)	433 (4.8)
Robbery, extortion (611–621)	137 (1.4)	26 (3.9)	111 (1.2)
Abduction, harassment (511–532)	120 (1.3)	13 (2.0)	107 (1.2)
Sexual offences (311–329)	86 (0.9)	18 (2.7)	68 (0.8)
Homicide (111–132)	34 (0.4)	20 (3.0)	14 (0.2)
Non-violent offence (ANZSOC code)	6211 (64.8)	342 (51.9)	5869 (65.7)
Offences against justice/government procedures (1511–1569)^a^	1424 (14.9)	91 (13.8)	1333 (14.9)
Theft (811–841)	1197 (12.5)	70 (10.6)	1127 (12.6)
Drug offences (1011–1099)	943 (9.8)	26 (3.9)	917 (10.3)
Public order offences (1311–1334)	818 (8.5)	43 (6.5)	775 (8.7)
Property/environmental damage (1211–1229)	671 (7.0)	24 (3.6)	647 (7.2)
Burglary (711)	337 (3.5)	56 (8.5)	281 (3.1)
Traffic offences (1411–1441)	318 (3.3)	17 (2.6)	301 (3.4)
Fraud, deception (911–999)	266 (2.8)	6 (0.9)	260 (2.9)
Weapons & explosives (1111–1129)	112 (1.2)	6 (0.9)	106 (1.2)
Miscellaneous offences (1611–1699)	125 (1.3)	3 (0.5)	122 (1.4)
Unknown	56 (0.6)	56 (8.5)	0

ANZSOC, Australian and New Zealand Standard Offence Classification.

a. Refers to breaches of custodial, community-based and violence and non-violence restraining orders and other technical offences against government/justice procedures.

Non-violent offending made up more than half of offences in people with a first diagnosis in prison (51.9% *v.* 39.6%) and two-thirds of those diagnosed in the community (65.7% *v.* 34.3%). However, people diagnosed with psychosis for the first time during a period of incarceration differed from those diagnosed in the community with regard to the type of offending (*P*<0.001) specially in committing drug-related offences (3.9% *v.* 10.3%), burglary (8.5% *v.* 3.1%), property and environmental damage (3.6% *v.* 7.2%), homicide (3.0% *v.* 0.2%) and robbery (3.9% *v.* 1.2%).

### Factors associated with a first diagnosis of psychosis in prison

Factors associated with being more likely to be first diagnosed with psychosis in prison compared with the community were: male gender, Aboriginal background, older age (25–34 years and 35–44 years), being from a disadvantaged area, and having at least one conviction for a violent offence prior to their first diagnosis (Table 3). Those with affective psychoses and substance-related psychoses and those who were not married (i.e. single/widowed/divorced/permanently separated or never reported their marital status) were less likely to be diagnosed in prison than the community. Offence type before the first diagnosis was not significant.

**Table 3:** Adjusted odds ratios (aOR)^a^ for factors associated with a first diagnosis of psychosis in prison

	Overall (*n* = 9588)			Men (*n* = 7342, 76.6%)			Women (*n* = 2246, 23.4%)		
	*n* (%)	aOR (95% CI)	*P*	*n* (%)	aOR (95% CI)	*P*	*n* (%)	aOR (95% CI)	*P*
Gender									
Women	2246 (23.4)	1		–	–		–	–	
Men	7342 (76.6)	2.27 (1.79–2.89)	<0.001	–	–	–	–	–	–
Aboriginality									
No	8036 (83.8)	1		6265 (85.3)	1		1771 (78.9)	1	
Yes	1552 (16.2)	1.81 (1.49–2.19)	<0.001	1077 (14.7)	1.65 (1.33–2.04)	<0.001	475 (21.1)	2.93 (1.85–4.64)	<0.001
Psychosis type									
Schizophrenia and related psychoses	5219 (54.4)	1		4084 (55.6)	1		1135 (50.5)	1	
Affective psychoses	840 (8.8)	0.46 (0.33–0.65)	<0.001	585 (8.0)	0.46 (0.32–0.66)	<0.001	255 (11.4)	0.49 (0.22–1.10)	0.084
Substance-related psychoses	3529 (36.8)	0.28 (0.22–0.35)	<0.001	2673 (36.4)	0.28 (0.22–0.36)	<0.001	856 (38.1)	0.30 (0.16–0.54)	<0.001
Age at first diagnosis									
<25	2864 (29.9)	1		2211 (30.1)	1		653 (29.1)	1	
25–34	2894 (30.2)	1.70 (1.37–2.11)	<0.001	2196 (29.9)	1.59 (1.26–2.01)	<0.001	698 (31.1)	2.74 (1.46–5.15)	0.002
35–44	2191 (22.9)	1.63 (1.29–2.06)	<0.001	1699 (23.1)	1.50 (1.17–1.93)	0.001	492 (21.9)	2.88 (1.46–5.68)	0.002
45+	1639 (17.1)	1.15 (0.87–1.52)	0.313	1236 (16.8)	1.13 (0.84–1.52)	0.416	403 (17.9)	1.29 (0.56–2.97)	0.546
SEIFA									
Advantaged	3283 (34.2)	1		2463 (33.5)	1		820 (36.5)	1	
Disadvantaged	5696 (59.4)	4.41 (3.42–5.69)	<0.001	4385 (59.7)	4.77 (3.60–6.33)	<0.001	1311 (58.4)	2.76 (1.50–5.08)	0.001
Unknown	609 (6.4)	1.14 (0.66–1.96)	0.633	494 (6.7)	1.31 (0.74–2.31)	0.350	115 (5.1)	0.41 (0.05–3.20)	0.396
Marital status									
Married (including de facto)	1119 (11.7)	1		795 (10.8)	1		324 (14.4)	1	
Other^b^	6079 (63.4)	0.75 (0.60–0.95)	0.015	4724 (64.3)	0.68 (0.53–0.87)	0.002	1355 (60.3)	1.42 (0.72–2.80)	0.308
Missing/unknown	2390 (24.9)	0.60 (0.45–0.80)	0.001	1823 (24.8)	0.52 (0.38–0.71)	<0.001	567 (25.2)	1.42 (0.64–3.16)	0.389
Offence type before diagnosis^c^									
Non-Violent	6267 (65.4)	1		4785 (65.2)	1		1482 (66.0)	1	
Violent	3321 (34.6)	1.07 (0.91–1.27)	0.406	2557 (34.8)	1.14 (0.95–1.37)	0.147	764 (34.0)	0.66 (0.40–1.09)	0.103

**SEIFA,:** Socio-Economic Indexes for Areas.

a. Adjusted by all variables in the table.

b. For example single, widowed, divorced, permanently separated.

c. Unknown offences (0.6%) were further adjusted for in the analysis.

In the gender-stratified analysis, a similar pattern to the overall findings was observed (Table 3). However, among women, affective psychoses and marital status was not significantly associated with a first diagnosis in prison.

### Time to a first diagnosis in prison

**Fig. 2:**
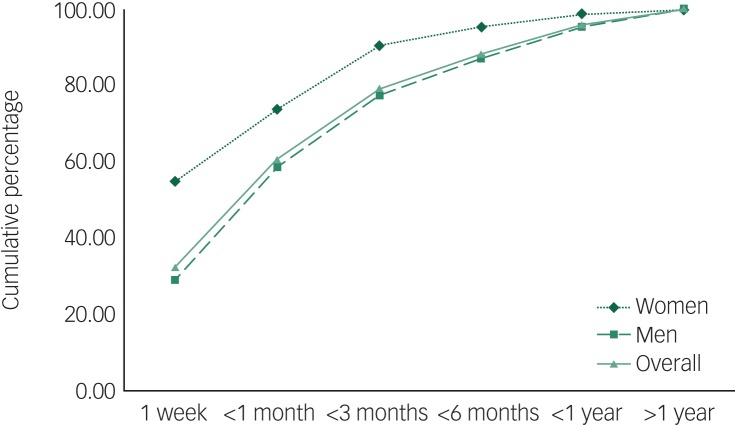
Time to a first diagnosis in prison – cumulative percentages for men and women.

**Fig. 3:**
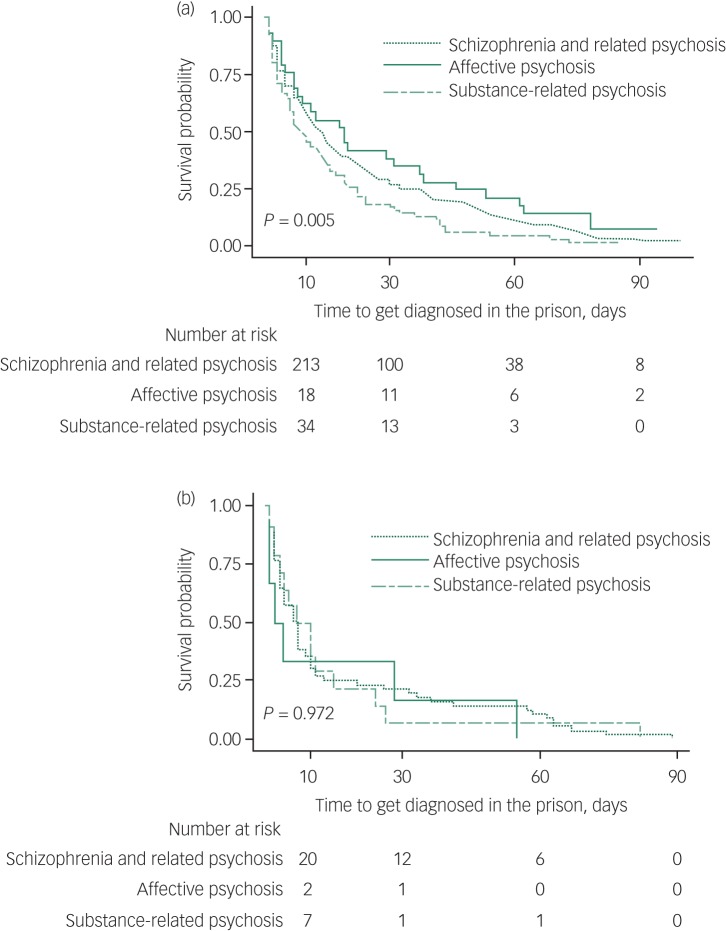
Time to a first diagnosis in prison – Kaplan-Meier survival curves by type of psychosis for (a) men and (b) women.

Among those diagnosed for the first time in prison (*n* = 659), over three-quarters (79.1%) were diagnosed within 3 months of imprisonment (Fig. 2). Overall women were diagnosed quicker compared with men. Further, more than one in every four men (29.0%) and more than half of all women (54.8%) were diagnosed within 1 week of incarceration. Proportionally more women (95.3%) than men (87.1%) were diagnosed within 6 months of imprisonment. Men with substance-related psychoses were diagnosed sooner after entry to prison than men with other psychoses, whereas no such differences were present in women (Fig. 3).

### Factors associated with diagnosis within 3 months of entry into prison

We also examined factors associated with early (within 3 months of incarceration) diagnosis of psychosis in prison (*n* = 659). Men were less likely to be diagnosed within 3 months compared with women (Table 4). In men, those with substance-related psychoses and those of unknown marital status were more likely to be diagnosed within 3 months of entry into prison. Although we found that being from a disadvantaged area and single marital status was significantly associated with an early diagnosis inside prison, overall, these effects were not significant among men. In women, factors associated with a diagnosis within 3 months included: disadvantaged and unknown SEIFA and unknown marital status.

**Table 4:** Adjusted hazard ratios (aHRs)^a^ for diagnosis of psychosis (within 3 months of imprisonment) among men and women

	Overall (*n* = 659)			Men (*n* = 575, 87.3%)			Women (*n* = 84, 12.7%)		
	*n* (%)	aHR (95% CI)	*P*	*n* (%)	aHR (95% CI)	*P*	*n* (%)	aHR (95% CI)	*P*
Gender									
Women	84 (12.7)	1		−	−		−	−	
Men	575 (87.3)	0.57 (0.44–0.74)	<0.001	−	−	−	−	−	−
Aboriginal									
No	486 (73.7)	1		440 (76.5)	1		46 (54.8)	1	
Yes	173 (26.2)	0.83 (0.68–1.02)	0.071	135 (23.5)	0.83 (0.66–1.04)	0.099	38 (45.2)	0.95 (0.57–1.58)	0.849
Psychosis type									
Schizophrenia and related psychoses	521 (79.1)	1		459 (79.8)	1		62 (73.8)	1	
Affective psychoses	40 (6.1)	1.16 (0.80–1.67)	0.437	33 (5.7)	1.14 (0.76–1.70)	0.530	7 (8.3)	1.83 (0.72–4.65)	0.206
Substance-related psychoses	98 (14.9)	1.38 (1.08–1.75)	0.009	83 (14.4)	1.49 (1.14–1.93)	0.003	15 (17.9)	0.96 (0.52–1.79)	0.903
Age at first diagnosis									
<25	154 (23.4)	1		139 (24.2)	1		15 (17.9)	1	
25–34	233 (35.4)	0.92 (0.72–1.17)	0.481	198 (34.4)	0.97 (0.75–1.25)	0.804	35 (41.7)	0.68 (0.35–1.31)	0.246
35–44	180 (27.3)	1.01 (0.77–1.31)	0.974	156 (27.1)	1.11 (0.84–1.48)	0.455	24 (28.6)	0.61 (0.29–1.26)	0.181
45+	92 (14.0)	1.12 (0.83–1.52)	0.461	82 (14.3)	1.14 (0.82–1.58)	0.445	10 (11.9)	1.05 (0.46–2.42)	0.900
SIEFA									
Advantaged	70 (10.6)	1		57 (9.9)	1		13 (15.5)	1	
Disadvantaged	572 (86.8)	1.43 (1.06–1.93)	0.021	502 (87.3)	1.33 (0.95–1.86)	0.098	70 (83.3)	2.18 (1.02–4.68)	0.045
Unknown	17 (2.6)	1.32 (0.71–2.46)	0.387	16 (2.8)	1.18 (0.61–2.29)	0.624	1 (1.2)	24.84 (2.59–237.88)	0.005
Marital status									
Married (including de facto)	116 (17.6)	1		105 (18.3)	1		11 (13.1)	1	
Other	440 (66.8)	1.31 (1.02–1.69)	0.032	385 (67.0)	1.22 (0.94–1.59)	0.132	55 (65.5)	2.15 (0.84–5.48)	0.109
Missing/unknown	103 (15.6)	2.21 (1.63–3.01)	<0.001	85 (14.8)	2.03 (1.46–2.82)	<0.001	18 (21.4)	3.85 (1.48–10.06)	0.006
Offence type before diagnosis									
Non-Violent	398 (60.4)	1		338 (58.8)	1		60 (71.4)	1	
Violent	261 (39.6)	0.92 (0.77–1.10)	0.366	237 (41.2)	0.89 (0.73–1.08)	0.229	24 (28.6)	1.17 (0.67–2.02)	0.579
Prior prison history									
No	492 (74.7)	1		419 (72.9)	1		73 (86.9)	1	
Yes	167 (25.3)	1.08 (0.87–1.34)	0.470	156 (27.1)	1.03 (0.82–1.30)	0.796	11 (13.1)	1.44 (0.67–3.09)	0.355

SIEFA, socio-economic indexes for areas.

a. Adjusted by all variables in the table.

### First diagnosis in the community and prior incarceration

Of those diagnosed with psychosis for the first time in the community setting (*n* = 37 830), 1.5% (*n* = 550) were diagnosed within 6 months of being released from prison (not presented in the tables). Of those, 62.9% were diagnosed within 3 months of release, and 29.5% within 1 month of release. The median time to diagnosis following release from prison was 2 months (IQR of 25 days to 4 months). Two-thirds were diagnosed following admission to hospital, 28.4% in emergency departments and 5.8% by community mental health services; 84.0% were male; and 31.6% were of Aboriginal heritage. Half were diagnosed with substance-related psychosis, 46.4% with schizophrenia and related psychosis and 3.6% with affective psychosis. A total of 60% were from disadvantaged areas. Median age at the time of being diagnosed in the community following release was 31 years (IQR = 25–39 years).

## Discussion

### Main findings

Overall, less than 2% (1.7%, *n* = 659) of those diagnosed for the first time with psychosis between 2006 and 2012 in NSW were diagnosed in prison. Importantly, this first diagnosis occurred soon after reception into prison with over half (60.5%) identified within 1 month, and more than three-quarters (79.1%) within 3 months. These findings suggest that effective systems are in place for identifying those with serious mental illness on entry to prison or soon thereafter. However, a significant number of individuals diagnosed in the community had passed through prison in the previous 6 months and not been diagnosed. Although it is reassuring that this vulnerable group is identified in a timely fashion following entry to prison, it may also suggest a need for screening closer to the time of release. It is also possible that greater coverage of the court liaison service that currently operates in 21 of the largest courts in NSW state-wide could pick up those identified soon after entry to prison. However, in more remote areas this may not be feasible from a cost perspective. According to our results, over half of those diagnosed inside prison had committed a non-violent act, which is more likely to attract a non-custodial sentence.

### Criminal convictions and a first diagnosis of psychosis

Our population-based study reported that one in every four (24.9%) people diagnosed for the first time with psychosis in NSW had a prior criminal conviction; this is consistent with a small UK cohort of study of patients with FEP (*n* = 301) of African–Caribbean ethnicity that reported that 23.6% had a criminal conviction prior to the onset of psychosis.^16^ Several studies have reported that criminal offending is associated with FEP, however, one systematic review and meta-analysis on FEP and violence also reported that severe injury to the victims from an act of a severe violence of the FEP group was not common.^17^ Our findings also suggest that over half of those who came in contact with the criminal justice system before their first diagnosis had committed non-violent offences including those diagnosed inside prison.

### Social determinants and a first diagnosis of psychosis

Our findings are also consistent with the theory of multiple trajectories to violent behaviour in those with psychosis and that the association between FEP and violence should not be attributed solely to psychosis-related factors.^18^ For example, factors commonly associated with increased risk of violence and offending in non-mentally ill populations (male gender, Aboriginality and coming from a disadvantaged area) that were associated with a first diagnosis of psychosis in prison in our study are also associated with increased risk for violence in both mentally ill and non-mentally ill populations.^19^ That most of those diagnosed with psychosis for the first time inside prison were detected soon after entry into prison also suggests that many of those with psychosis and at risk of offending may not be identified by mental health services in the community. Reasons for this include coming from a demographic that avoids mental health contact because of the stigma this may promote or a lack of availability and/or access to mental health services by those who are disadvantaged.

A first diagnosis of psychosis occurred earlier in women than men in prison (73.8% within 1 month and 90.5% within 3 months *v.* 58.6% within 1 month and 77.4% within 3 months) suggesting better access to services for women than men in the correctional system or a greater willingness by women to report symptoms of mental illness at time of entry. According to the 2009 NSW Inmate Health Survey, a gender disparity exists in receiving a mental health assessment or treatment with 54% of women reporting having ever received a mental health assessment compared with 47% of men.^20^ In our study, women were equally as likely to be diagnosed in the community as men (99.5% *v.* 97.3%). However, men were 2.27 times more likely to be diagnosed inside prison compared with women.

The proportion of Aboriginal people diagnosed for the first time in prison was higher than non-Aboriginal (6.9% *v.* 1.4%). Those with Indigenous heritage were more likely to be diagnosed for the first time in prison rather than in the community, which also supports the previous findings on ethnicity disparities in regards to mental health services available in the community.^21^ Not surprisingly, those from disadvantaged areas were more likely to be diagnosed for the first time in prison rather than in the community, suggesting a lack of adequate pathways to care and potentially limited legal avenues to facilitate diversion away from custody into treatment on the grounds of mental health.^22^ Another factor that is independent of the availability of mental health services could be the mechanism via which individuals come into contact with mental health services. In prison every arrival is screened and triaged for possible mental health assessment and this is often done with the benefit of observations made by police and other factors in the justice system regarding the individual's presentation and behaviour. The services in the community could be the same but there is no universal mandatory assessment process like there is in prison.

### Limitations

Limitations of this study include no historical data being available prior to 2001 and hence we could not establish the exact time of onset of psychosis. Limitations also include the lack of data availability for those presenting to the emergency departments before January 2005. Another limitation is that the cohort is inherently biased towards those with more severe mental illness as it was defined by admission to hospital or emergency department presentation. The study was conducted in NSW only and it is possible that some individuals may have been previously diagnosed interstate or overseas. Similarly, those diagnosed in private clinics or treated by general practitioners were not accounted for in this study. However, most mentally ill people with psychosis are treated by the NSW public mental health services.

### Implications

It is likely that some of those diagnosed with psychosis for the first time in prison were identified as mentally unwell even quicker on entry to prison and that a time lag occurred between identification by nursing staff (who conduct the health reception assessment in NSW prisons) and subsequent diagnostic confirmation by a psychiatrist that would yield the diagnosis date in the administrative data collections we examined. Further research is needed to follow-up individuals diagnosed for the first time in prison to investigate whether appropriate treatment, including access to pharmacotherapies, was received. One study of 18 185 USA prisoners found that more than half who were taking medication for a mental health condition on admission to prisons did not receive pharmacotherapy during incarceration.^23^ Further work needs to be done in the context of the current study to determine medication continuity for this population in both directions – on both entry to prison from the community and on release. The aspirational model of prison healthcare is that of ‘equivalence’, which suggests that the same standard of treatment in the community should be available to those citizens in prison. And vice versa, treatment initiated in prison should be continued by mental health services in the community.

We recently reported that receiving a treatment order in the courts under the Mental Health Act had a positive impact in terms of reoffending and that an increased number of mental health treatment episodes was related to a reduced risk of reoffending.^24^ This finding is consistent with a UK study of 1717 individuals in prison with psychosis that reported that treatment in prison was associated with delayed time to reoffending after release from prison.^25^ Further work is needed to determine whether those with psychosis identified soon after entry to prison may have committed serious crimes precluding diversionary alternatives to custody or whether system issues such as no diversionary options available at smaller courts may be responsible for this.

It is well-established that those with serious mental illness face difficulties following release from prison such as homelessness.^26^ The risk of suicide and drug overdose death have been found to be high among newly released prisoners.^27^ Connecting those with mental illness with community-based services on release from prisons is clearly an important component of successful reintegration and avoiding post-release mortality.

## Data Availability

We have full access to the study data. Our data is associated with a manuscript available at https://doi.org/10.1192/bjo.2018.71.
